# Relationship of autonomic imbalance and circadian disruption with obesity and type 2 diabetes in resistant hypertensive patients

**DOI:** 10.1186/1475-2840-10-24

**Published:** 2011-03-22

**Authors:** Leandro Boer-Martins, Valéria N Figueiredo, Caroline Demacq, Luiz C Martins, Fernanda Consolin-Colombo, Márcio J Figueiredo, Fernando PS Cannavan, Heitor Moreno

**Affiliations:** 1 Cardiovascular Pharmacology Laboratory, Faculty of Medical Sciences and Clinic Hospital, State University of Campinas (UNICAMP), Campinas, São Paulo, Brazil; 2 Cardiology Department, Faculty of Medical Sciences and Clinic Hospital, State of Campinas (UNICAMP), Campinas, São Paulo, Brazil; 3 Hypertension Unit, Heart Institute (InCor), Clinical Hospital of São Paulo, Faculty of Medicine, University of São Paulo, São Paulo, Brazil; 4 Cardiovascular & Metabolism Unit, Pharma Sector, Novartis Biociências S.A., São Paulo, Brazil

## Abstract

**Background:**

Hypertension, diabetes and obesity are not isolated findings, but a series of interacting interactive physiologic derangements. Taking into account genetic background and lifestyle behavior, AI (autonomic imbalance) could be a common root for RHTN (resistant hypertension) or RHTN plus type 2 diabetes (T2D) comorbidity development. Moreover, circadian disruption can lead to metabolic and vasomotor impairments such as obesity, insulin resistance and resistant hypertension. In order to better understand the triggered emergence of obesity and T2D comorbidity in resistant hypertension, we investigated the pattern of autonomic activity in the circadian rhythm in RHTN with and without type 2 diabetes (T2D), and its relationship with serum adiponectin concentration.

**Methods:**

Twenty five RHTN patients (15 non-T2D and 10 T2D, 15 males, 10 females; age range 34 to 70 years) were evaluated using the following parameters: BMI (body mass index), biochemical analysis, serum adiponectinemia, echocardiogram and ambulatory electrocardiograph heart rate variability (HRV) in time and frequency domains stratified into three periods: 24 hour, day time and night time.

**Results:**

Both groups demonstrated similar characteristics despite of the laboratory analysis concerning T2D like fasting glucose, HbA1c levels and hypertriglyceridemia. Both groups also revealed disruption of the circadian rhythm: inverted sympathetic and parasympathetic tones during day (parasympathetic > sympathetic tone) and night periods (sympathetic > parasympathetic tone). T2D group had increased BMI and serum triglyceride levels (mean 33.7 ± 4.0 *vs *26.6 ± 3.7 kg/m^2 ^- p = 0.00; 254.8 ± 226.4 *vs *108.6 ± 48.7 mg/dL - p = 0.04), lower levels of adiponectin (6729.7 ± 3381.5 *vs *10911.5 ± 5554.0 ng/mL - p = 0.04) and greater autonomic imbalance evaluated by HRV parameters in time domain compared to non-T2D RHTN patients. Total patients had HRV correlated positively with serum adiponectin (r = 0.37 [95% CI -0.04 - 1.00] p = 0.03), negatively with HbA1c levels (r = -0.58 [95% CI -1.00 - -0.3] p = 0.00) and also adiponectin correlated negatively with HbA1c levels (r = -0.40 [95% CI -1.00 - -0.07] p = 0.02).

**Conclusion:**

Type 2 diabetes comorbidity is associated with greater autonomic imbalance, lower adiponectin levels and greater BMI in RHTN patients. Similar circadian disruption was also found in both groups indicating the importance of lifestyle behavior in the genesis of RHTN.

## Background

Hypertension, diabetes and obesity are not isolated findings, but a series of interactive physiologic derangements [1]. For instance, it is well known that obesity and diabetes mellitus are factors associated with resistance to antihypertensive drugs. An understanding of interactions among these pathophysiologic pathways can assist in choosing treatment and thereby improving total cardiovascular risk management [1].

Autonomic imbalance, characterized by a hyperactive sympathetic system and a hypoactive parasympathetic system, is associated with various pathological conditions [2,3]. Over time, excessive energy demands on the system can lead to premature aging and diseases [2,3]. Therefore, autonomic imbalance may be a final common pathway to increased morbidity and mortality from a host of conditions and diseases, including cardiovascular disease [4,5].

Heart rate variability (HRV) may be used to assess autonomic imbalances, diseases and mortality [6]. Measures of heart rate variability (HRV) in both time and frequency domains have been used successfully to index vagal activity [7]. Nevertheless, while there are some differences among HRV parameters found in many studies, the consensus is that lower values of these indices of vagal function are associated prospectively with death and disability [8]. Parasympathetic activity and HRV have been associated to immune dysfunction and inflammation, which have been implicated in a wide range of conditions including CVD and diabetes [2,3].

There is a pathogenic link between autonomic imbalance and insulin resistance and hypertension onset [9-14]. In addition to genetic background and environment, AI (autonomic imbalance) could be a common root of HTN (hypertension) or HTN plus T2D (type 2 diabetes) comorbidity development. T2D comorbidity can be added to HTN by decreased energy dissipation, gaining weight and then insulin resistance [15]. It is known that a chronic increase in sympathetic outflow has been reported to decrease β-adrenergic responsiveness itself, by a down-regulation of β-adrenergic receptors, which are known to mediate energy expenditure either at rest or after food intake [16].

These obesity-related disorders including metabolic syndrome, diabetes, atherosclerosis, hypertension, and coronary artery disease are associated with dysregulated adipokine(s) expression such as adiponectin [17].

Adiponectin is a hormone that is produced by adipocytes [18]. In patients with type 2 diabetes mellitus, low plasma adiponectin levels are associated with insulin resistance and have also been shown to be an independent predictor of type 2 diabetes mellitus [19]. In addition, sympathetic nervous overactivity is associated with hypoadiponectinemia [20,21]. However, there is still limited information on the relationship between plasma adiponectin, obesity, T2D and cardiac autonomic nervous function, especially in resistant hypertension (RHTN).

In order to better understand the triggered emergence of obesity and T2D comorbidity in resistant hypertension, we investigated the pattern of autonomic activity in the circadian rhythm in this population with and without type 2 diabetes (T2D) and its relationship with serum adiponectin concentration.

## Methods

Twenty-five (25) RHTN subjects [22] [15 non-T2D and 10 T2D, 15 (60%) females and 10 (40%) males], regularly followed in the ambulatory service of cardiovascular clinical pharmacology, complying with pharmacological prescription for HTN and T2D, were recruited to participate in this transversal study. The diagnosis of resistant hypertension required a good office blood pressure measurement technique and ambulatory blood pressure monitoring (ABPM) to confirm persistently elevated blood pressure levels [23]. Pseudoresistance cases, including lack of blood pressure control secondary to poor medication adherence, were properly observed and excluded [24]. White coat hypertension (WCH) was excluded by ABPM [23]. Regarding obstructive sleep apnea (OSA), only patients classified as "low risk" by Berlin sleep questionnaire were enrolled [25]. Resistant hypertension include patients whose blood pressure is uncontrolled with use of more than three medications or patients whose blood pressure is controlled, but required four or more medications to achieve blood pressure goals [23]. All subjects provided written informed consent and the study was approved by the local ethics committee.

The exclusion criteria comprised: acute or moderate-severe renal dysfunction, non-complied pharmacological prescription, use of beta-blockers within the last six months, severe obesity (body mass index ≥ 35 kg/m^2^), heart failure (ejection fraction < 50%), valvular heart disease, cardiomyopathies, primary hyperaldosteronism [aldosterone:PRA ratio > 20 ng per 100 mL per ng.ml(-1)h(-1)], sleep apnea (classified as "high risk" by the Berlin sleep questionnaire), atrial fibrillation, sick sinus syndrome, supraventricular and ventricular tachycardias, aortic disease (Marfan's syndrome, coarctation of the aorta, aneurysms or aortic surgery, etc), history of coronary artery disease or proven coronary artery disease by coronary angiography or noninvasive tests, familial hyperlipidemia, asthma or chronic obstructive lung disease, pregnancy or oral contraceptive use, connective tissue disorders, neurological problems, malignancies, psychiatric diseases, other than T2D endocrinological diseases, smoking, alcohol use and drug abuse.

### Blood pressure measurements

Blood pressure was assessed by considering the orientations of the last guideline on hypertension of the European Society of Cardiology [26]. Blood pressure (SBP - systolic blood pressure/DBP - diastolic blood pressure) was measured three times for each subject using a digital sphygmomanometer (Omron HEM-711DLX) on the right upper arm in the sitting position after a 10-minute rest. The average of two consecutive measurements was used with a variation lower than 5 mmHg.

### Laboratory analysis

All subjects underwent the following laboratory tests: hemogram, serum fasting glucose, glycolized hemoglobin (HbA1c), serum urea and creatinine, serum total cholesterol, serum LDL-cholesterol fraction, serum HDL-cholesterol fraction, serum triglycerides, serum uric acid, serum sodium and potassium, and plasma adiponectin levels (Quantikine^® ^Human total adiponectin/Acrp30 Immunoassay DRP 300, R&D Systems, Inc., Minneapolis, USA).

### Echocardiographic examination

All subjects were submitted to standard transthoracic echocardiography in the left lateral decubitus position by using a Vivid 7 Pro machine with a 2.5 Mhz probe (General Electric, Florida, USA). Standard transthoracic views were used to determine end-diastolic and end-systolic volumes, stroke volume index, left ventricular ejection fraction (LVEF), transmitral E and A waves velocities, E/A ratio, tissue doppler velocity of the mitral annulus and left ventricular mass index (LVMI). The left ventricular diastolic dysfunction (LVDD) was assessed by the Omnen SR and Nishimura RA algorithm[27]. The echocardiographic examination was performed by only one experienced cardiologist examiner. There was no intra or inter-observer measurement variability.

### Heart rate variability

Heart rate variability (HRV) parameters were derived from the recording of 24-hour Holter monitoring and analyzed in time and frequency domains. Measures were stratified into three time periods for time domain: 24 hour period (24 h), day time period (DT), 1 p.m. to 5 p.m. and night time period (NT), from 2 a.m to 6 a.m. Frequency domain measures were stratified into two periods of one hour each at 3 a.m. (night time period - NT) and 3 p.m. (day time period - DT). A three-channel, 24-hour Holter recording was obtained from each subject using the Cardio light digital 24-hour recorder device and the CardioSmart Institutional CS 550 software (Cardio Sistema Comércio e Indústria Ltda, São Paulo, SP, Brazil).

Time domain HRV parameters included the following measures [6,28]:

- rMSSD (ms): Square root of the mean of the squares of differences between successive RR intervals.

- SDNN (ms): Standard deviation of all normal RR intervals in 24-hour Holter recording.

- SDANN (ms): Standard deviation of RR intervals means in all 5-minute segments of 24-hour recording.

- pNN50 (%): Percentage of differences between successive RR intervals that are greater than 50 ms.

Frequency domain measures were calculated using the Fast Fourier Transform (FFT) to break down the time series to its underlying periodic function. Frequency domain HRV parameters included the following measures [6,28]:

- Low frequency (LF) and high frequency (HF) measured in normalized units, which represent the relative value of each power component in proportion to the total power minus the very low frequency (VLF) component. Normalized LF (LF nu) was calculated as LF power in normalized units LF/(total power-VLF) × 100, and normalized HF (HF nu) as HF power in normalized units HF/(total power-VLF) × 100. Low frequency (LF) and high frequency (HF). LF nu and HF nu denote the energy in the heart period power spectrum between 0.04 and 0.15 Hz (which is due to the joint action of the vagal and sympathetic components on the heart, with a predominance of the sympathetic ones) and 0.15 and 0.40 Hz (which corresponds to the respiratory modulation and is an indicator of the performance of the vagus nerve on the heart), respectively. "Day time" and "night time" were established at 3:00 p.m. and 3:00 a.m., respectively, in order to collect HRV data during wake and sleep periods.

### Statistical analysis

Data were expressed as mean (μ) and standard deviation (SD) or mean (μ) and standard error of the mean (SEM) for HRV measures. Unpaired groups were compared using Mann-Whitney U test while correlation analysis were performed using Spearman's rank test. Fisher's exact test was used to determine whether a certain group had significantly different proportion of a particular characteristic. The level of statistical significance accepted was less than 0.05. All data were entered into a spreadsheet program (MS Excel Microsoft Corp, Phoenix, Arizona, USA) for statistical analysis. Analytical statistics were performed by Analyse-it version 2.21 Excel 12+ (Analyse-it Software Ltd., Leeds, UK), a statistical add-in program for Excel (MS Excel Microsoft Corp, Phoenix, Arizona, USA).

## Results

**Figure 1:**
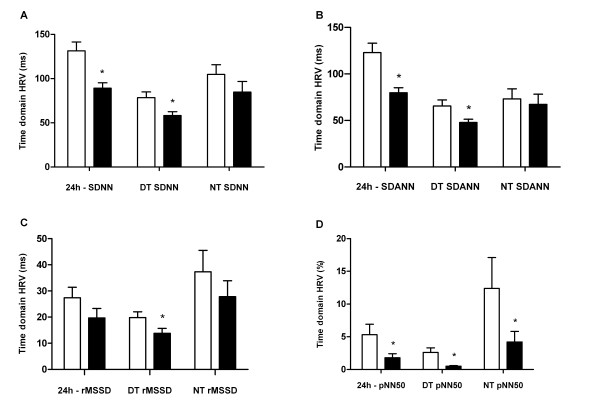
**Autonomic imbalance**. Heart rate variability (HRV) in time domain between non type 2 diabetes (T2D) (white column) and T2D (black column) RH patients. A: SDNN (ms): Standard deviation of all normal RR intervals in 24-hour Holter recording; B: SDANN (ms): Standard deviation of RR means intervals in all 5-minute segments of 24-hour recording; C: rMSSD (ms): Square root of the mean of the squares of differences between successive RR intervals; D: pNN50 (%): Percentage of differences between successive RR intervals that are greater than 50 ms; DT: Day time (1 p.m. - 5 p.m.); NT: Night time (2 a.m. - 6 a.m.); 24 h: 24 hours; (*) Statistical significance (p < 0.05).

The general characteristics of the study groups are listed in table 1. No statistical differences were observed between the non-T2D and T2D subgroups with respect to age and gender. The mean ages were 54.7 and 54.9 in non-T2D and T2D patients, respectively. Women made up equally 40% of the patients in these groups. Both groups demonstrated similar characteristics despite of the laboratory analysis concerning T2D diagnosis like fasting glucose (167.8 ± 9.2 vs 92.9 ± 9.2 mg/dL - p < 0.0001) and HbA1c levels (9.3 ± 2.1 vs 5.8 ± 0.3% - p < 0.0001) (Table 1). However, the T2D group showed a greater BMI and higher serum triglyceride levels than the non-T2D group (33.7 ± 4.0 vs 26.6 ± 3.7 kg/m^2^, - p = 0.0002; 254.8 ± 226.4 vs 108.6 ± 48.7 mg/dL - p = 0.041) (Table 1). Concerning HRV parameters, the following evaluations were reduced in T2D: 24 hour-SDNN (89.1 ± 19.9 *vs *122.9 ± 39.5 ms; p = 0.0009), Day time SDNN (58.2 ± 13.6 *vs *78.5 ± 24.9 ms; p = 0.03), 24 hour-SDANN (79.8 ± 17.1 *vs *122.9 ± 39.5 ms; p = 0.0012), Day time SDANN (47.8 ± 3.5 *vs *65.5 ± 6.5 ms; p = 0.03), Day time rMSSD (13.8 ± 1.9 *vs *19.8 ± 2.2; p = 0.05), 24 hour-pNN50 (1.8 ± 2.1 vs 5.3 ± 6.4%; p = 0.047), Day time pNN50 (0.5 ± 0.5 *vs *2.6 ± 2.9%; p = 0.035) and Night time pNN50 (4.2 ± 1.6 *vs *12.4 ± 4.7;p = 0.04) (Figure 1). Although the remaining HRV parameters in time domain have demonstrated greater autonomic imbalance in the T2D group, they did not achieve statistically significance (p > 0.05) (Figure 1).

**Table 1:** General characteristics of study groups

Characteristic/Variable	Non-T2D group (n = 15)	T2D group (n = 10)	*p*-value
Gender	60% (female)/40% (male)	60% (female)/40% (male)	1.00
Age (year)	54.7 ± 10.0 [49.2 - 60.3]	54.9 ± 8.7 [48.7 - 61.1]	0.89
Hemoglobin (g/dL)	13.6 ± 1.1 [13.0 - 14.3]	13.6 ± 1.4 [12.6 - 14.6]	0.97
Hematocrit (%)	41.1 ± 2.7 [39.6 - 42.7]	40.4 ± 4.0 [37.5 - 43.3]	0.56
Body mass index (kg/m^2^) *	26.6 ± 3.7 [24.5 - 28.7]	33,7 ± 4,0 [30.8 - 36.6]	**0.00**
Fasting glucose (mg/dL) *	92.9 ± 9.2 [87.9 -98.0]	167.8 ± 64.0 [117.8 - 258.2]	**0.00**
HbA1c (%) *	5.8 ± 0.3 [5.6 - 6.0]	9.3 ± 2.1 [7.7 - 10.8]	**0.00**
Serum adiponectin (ng/mL) *	10911.5 ± 5554.0 [7835.7 - 13987.2]	6729.7 ± 3381.5 [4310.8 - 9148.7]	**0.04**
Serum urea (mg/dL)	34.2 ± 12.8 [27.1 - 41.3]	38.0 ± 7.8 [32.4 - 43.6]	0.46
Serum creatinine (mg/dL)	0.9 ± 0.2 [0.8 - 1.0]	0,9 ± 0.1 [0.7 - 1.0]	0.93
Total cholesterol (mg/dL)	193.7 ± 48.8 [166.7 - 220.8]	193.5 ± 33.2 [169.8 - 217.2]	0.36
LDL-cholesterol (mg/dL)	119.4 ± 47.0 [93.4 - 145.4]	104.3 ± 31.1 [82.0 - 126.6]	0.84
HDL-cholesterol (mg/dL)	50.8 ± 17.0 [41.4 - 60.2]	42.9 ± 11.0 [35.0 - 50.8]	0.21
Serum triglycerides (mg/dL) *	108.6 ± 48.7 [81.6 -135.6]	254.8 ± 226.4 [92.9 - 416.7]	**0.04**
Uric Acid (mg/dL)	5.3 ± 1.1 [4.7 - 6.0]	5.7 ± 1.2 [4.8 - 6.5]	0.60
Na (mEq/L)	140.1 ± 2.2 [138.9 - 141.3]	138.8 ± 2.0 [137.3 - 140.3]	0.17
K (mEq/L)	4.3 ± 0.5 [4.0 -4.6]	4.2 ± 0.4 [3.8 - 4.5]	0.49
UACR (mg/g)	11.3 ± 13.2 [4.0 - 18.7]	138.2 ± 229.8 [-26.2 - 302.6]	0.17
Office SBP (mmHg)	154.3 ± 21.6 [142.3 -166.2]	156.5 ± 30.6 [134.7 - 178.4]	0.93
Office DBP (mmHg)	92.9 ± 10.8 [86.9 - 98.8]	90.1 ± 18.2 [77.0 - 103.1]	0.33

The values are expressed as means ± standard deviation; [95% confidence interval]; (*) Statistical significance (p < 0.05); Non-T2D: non type 2 diabetes resistant hypertension; T2D: type 2 diabetes resistant hypertension.

**Figure 2:**
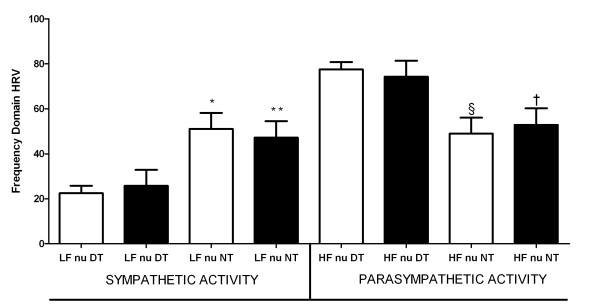
**LF nu: Circadian disruption**. Heart rate variability (HRV) in frequency domain between non type 2 diabetes (T2D) (white column) and T2D (black column) RH patients. LF nu: Low frequency in normalized units; HF nu: High frequency in normalized units; DT: Day time (3 p.m.); NT: Night time (3 a.m.); Statistical significance: (*) p < 0.05 *vs *LF nu DT non-T2D (**) p < 0.05 *vs *LF nu DT T2D; (§) p < 0.05 *vs *HF nu DT non-T2D; (†) p < 0.05 *vs *HF nu DT T2D.

Frequency domain parameters demonstrated inverted pattern of tone intensity for both branches of the autonomic system during day and night periods in both groups. Regarding the non-T2D group, LF nu during day and night were 22.5 ± 3.2 *vs *51.0 ± 7.1 (p = 0.00) and HF nu were 77.5 ± 3.2 *vs *48.9 ± 7.1 (p = 0.00), respectively. For the same group, HF nu during day and night were 77.5 ± 3.2 *vs *48.9 ± 7.1 (p = 0.00), respectively. Regarding the T2D group, LF nu during day and night were 25.7 ± 7.1 *vs *47.1 ± 7.3 (p = 0.01) and HF nu were 74.2 ± 7.1 *vs *52.8 ± 7.3 (p = 0.01), respectively (Figure 2). For the same group, HF nu during day and night were 74.2 ± 7.1 *vs *52.8 ± 7.3 (p = 0.01), respectively (Figure 2). There were no differences between non-T2D and T2D groups in frequency domain parameters (Table 2).

**Table 2:** Heart rate variability (HRV) in time and frequency domain between non-T2D and T2D resistant hypertension patients

HRV variable	Non-T2D group (n = 15)	T2D group (n = 10)	*p*-value
24 hour-SDNN *	131.5 ± 9.9 [110 - 152,9]	89.1 ± 6.2 [74.9 - 103.3]	**0.00**
Day time SDNN *	78.5 ± 6.4 [64.7 - 92.3]	58.2 ± 4.3 [48.5 - 67.9]	**0.03**
Night time SDNN	104.7 ± 11.1 [80.8 -128.5]	84.7 ± 12.0 [57.6 - 111.8]	0.21
24 hour-SDANN *	122.9 ± 10.2 [101.0 - 144.8]	79.8 ± 5.4 [67.6 - 92.0]	**0.00**
Day time SDANN *	65.5 ± 6.5 [51.5 - 79.4]	47.8 ± 3.5 [39.7 - 55.9]	**0.03**
Night time SDANN	73.2 ± 10.7 [50.1 - 96.3]	67.3 ± 11.0 [42.3 - 92.3]	0.80
24 hour-pNN50 *	5.3 ± 1.6 [1.8 - 8.9]	1.8 ± 0.6 [0.3 - 3.3]	**0.05**
Day time pNN50 *	2.6 ± 0.7 [0.9 - 4.2]	0.5 ± 0.1 [0.1 - 0.8]	**0.04**
Night time pNN50 *	12.4 ± 4.7 [2.3 - 22.5]	4.2 ± 1.6 [0.5 - 7.9]	**0.04**
24 hour-rMSSD	27.4 ± 4.0 [18.7 - 36.1]	19.7 ± 3.6 [11.6 - 27.8]	0.17
Day time rMSSD *	19.8 ± 2.2 [14.9 - 24.7]	13.8 ± 1.9 [9.5 - 18.1]	**0.05**
Night time rMSSD	37.3 ± 8.2 [19.6 - 55.0]	27.8 ± 6.1 [14.0 - 41.6]	0.21
Day time LF nu	22.5 ± 3.2 [15.4 - 29.5]	25.7 ± 7.1 [9.6 - 41.8]	0.97
Night time LF nu	51.0 ± 7.1 [35.8 - 66.3]	47.1 ± 7.3 [30.5 - 63.8]	0.80
Day time HF nu	77.5 ± 3.2 [70.4 - 84.5]	74.2 ± 7.1 [58.1 - 90.3]	0.97
Night time HF nu	48.9 ± 7.1 [33.6 - 64.1]	52.8 ± 7.3 [36.2 - 69.4]	0.80

The values are expressed as means ± standard error of the mean; [95% confidence interval]; (*) Statistical significance (p < 0.05). SDNN (ms): Standard deviation of all normal RR intervals in 24-hour Holter recording; SDANN (ms): Standard deviation of means of RR intervals in all 5-minute segments of 24-hour recording; pNN50 (%): Percentage of differences between successive RR intervals that are greater than 50 ms; rMSSD (ms): Square root of the mean of the squares of differences between successive RR intervals; LF nu: Low frequency in normalized units from the power spectra of HRV by computer analysis using Fast Fourier Transformation (FFT); HF nu: High frequency in normalized units from the power spectra of HRV by computer analysis using FFT; Non-T2D: non type 2 diabetes resistant hypertension; T2D: type 2 diabetes resistant hypertension.

**Figure 3:**
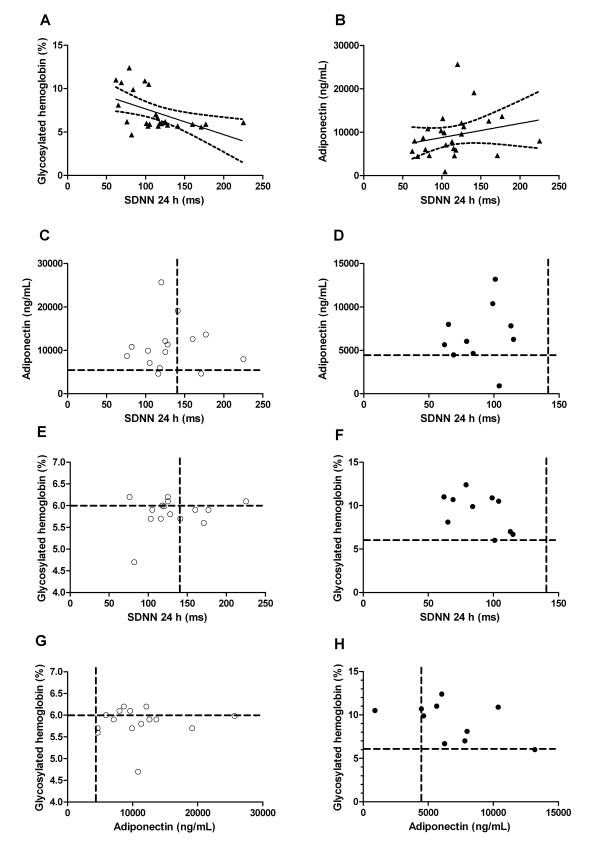
**A: Correlation between HRV and glycosylated hemoglobin (HbA1c) levels of total RH patients (r = -58; [95% CI -1.0 - -0.3]; p = 0.00); B: Correlation between HRV and adiponectin of total RH patients (r = 0.37; [95% CI -0.04 - 1.0]; p = 0.03); C and D: Matrix plot of the distribution of values between HRV and adiponectin; E and F: Matrix plot of the distribution of values between HRV and HbA1c levels; G and H: Matrix plot of the distribution between HbA1c levels and adiponectin**. Non-T2D group: white circles; T2D group: black circles. Parameters for the matrix plot in means: Adiponectin (5548 ng/mL - Healthy volunteers evaluated with plasma EDTA of Quantikine^® ^lab test), SDNN 24 h (141 ms)[6] and HbA1c (6.0%).

Total patients (non-T2D and T2D groups) had HRV correlated positively with serum adiponectin (r = 0.37 [95% CI -0.04 - 1.00] p = 0.03) and negatively with HbA1c levels (r = -0.58 [95% CI -1.00 - -0.3] p = 0.00). Total patients had also adiponectin correlated negatively with HbA1c levels (r = -0.40 [95% CI -1.00 - -0.07] p = 0.02) (Figure 3).

It has also been observed that the T2D group was taking more anti-hypertensive drugs than the non-T2D group (Table 3). In addition, the T2D group demonstrated greater prevalence of left ventricular diastolic dysfunction than the non-T2D group despite of similar left ventricle hypertrophy index (Table 4).

**Table 3:** Anti-hypertensive (anti-HTN) drugs distribution

Characteristic/Variable	Non-T2D group (n = 15)	T2D group (n = 10)	*p*-value
Total anti-HTN drugs*	3.3 ± 0.5 [3.1 - 3.6]	4.1 ± 0.7 [3.6 - 4.6]	**0.02**
Thiazide diuretic	100% (15)	100% (10)	-
Aldosterone receptor inhibitor	26.6% (4)	50% (5)	0.40
Angiotensin converting enzyme inhibitor	66.6% (10)	50% (5)	0.68
Angiotensin receptor blocker	40% (6)	70% (7)	0.23
Calcium channel blocker	86.6% (13)	100% (10)	0.50
Centrally acting anti-hypertensive drug	6.6% (1)	40% (4)	0.12

The values are expressed as means ± standard deviation; [95% confidence interval]; (*) Statistical significance (p < 0.05). Non-T2D: non type 2 diabetes resistant hypertension; T2D: type 2 diabetes resistant hypertension.

**Table 4:** Echocardiographic parameters of study groups

Characteristic/Variable	Non-T2D group (n = 15)	T2D group (n = 10)	*p*-value
LVEF (%)	70.3 ± 7.3 [66.3 -74.4]	66.5 ± 21.7 [51.0 - 82.0]	0.28
LVMI (g/m^2^)	134.1 ± 30.9 [116.9 - 151.2]	148.4 ± 31.6 [125.8 - 171.0]	0.31
LVDD	Present in 60%	Present in 100%	0.05

The values are expressed as means ± standard deviation; [95% confidence interval]; Non-T2D: non type 2 diabetes resistant hypertension; T2D: type 2 diabetes resistant hypertension; LVEF: left ventricle ejection fraction; LVMI: left ventricle mass index; LVDD: left ventricle diastolic dysfunction (assessed by the Omnen SR and Nishimura RA algorithm[27]).

## Discussion

### Important findings

We found that the AI is more impaired in T2D RHTN patients than the non-T2D group. As expected, the T2D group had greater BMI and serum triglycerides than the non-T2D group. Considering the total of patients, an established negative correlation between HbA1c levels and serum adiponectin concentration was also observed. In addition to these well-known characteristics between non-T2D and T2D patients, considering the total patients, HRV correlated positively with adiponectin and negatively with HbA1c levels. Interestingly, despite of the higher AI in the T2D subgroup, both groups demonstrated similar inverted pattern of sympathetic and parasympathetic tones during day and night periods. As far as we know, this is the first time that AI and circadian disruption (CD) were evaluated in RHTN patients with or without T2D in order to better understand their synergistic role in obesity and T2D association with RHTN.

### Historical aspects of autonomic dysfunction

Since the fifth decade, cardiovascular autonomic researchers have cogitated whether AI would be a cause or consequence of hypertension [29]. Subsequently, it was demonstrated that AI could lead not only to hypertension, but also to T2D [7]. Reasonable amount of data provide evidences for prediction hypertension or diabetes onset due autonomic imbalance evaluated by HRV parameters in time or frequency domain [9-14]. Autonomic imbalance causes, at first, increased insulin sensitivity and reduced energy dissipation [15]. Concomitantly, insulin resistance impairs the overall mean levels of cardiac autonomic modulation among persons with T2D [30].

### Pathogenesis of autonomic dysfunction and metabolic disorders

The increased concentration of angiotensin II inhibits intracellular signaling to GLUT 4 expression on the surface of the cells leading to insulin resistance [31,32]. However, the observation that not all subjects follow this link suggests that other factors, including genetic predisposition and environment, can influence it at various levels [33-38].

A chronic increase in sympathetic outflow has been reported to decrease β-adrenergic responsiveness itself, through down-regulation of β-adrenergic receptors, which are known to mediate energy expenditure both at rest and after food intake [16]. This mechanism could result in a reduced ability to dissipate energy leading to weight gain. At first glance, it appears to be in contrast to the previous reports in rats, where the down-regulation of β-adrenergic receptors, following chronic catecholamine stimulation, has been associated with increased insulin sensitivity [39]. Moreover, the hypothalamus is a regulatory center of satiety and of the autonomic nervous system (ANS). Therefore, abnormalities in the hypothalamus may cause obesity and autonomic dysfunction. This may explain the alterations observed in the HRV indices [40].

Corroborating the mechanism above, the correlation between high sympathetic activity and hypoadiponectinemia was previously established [21]. Since we evaluated RHTN patients and hypoadiponectinemia is also associated with sympathetic activation and severity of OSA [41], only true RHTN patients classified as "low risk" (Berlin sleep questionnaire) of OSA diagnosis were included in this study. We have found positive correlation between HRV and adiponectinemia and it is properly presumable to us that this adipocytokine may play an important role in the development of obesity and T2D in RHTN by the AI patient status. Recently, an interventional clinical trial with prolonged insulin-glucose infusion was performed with healthy human subjects in order to evaluate the effect of insulin on adiponectin multimers and nuclear factor-kappaB (NF-kB) activity in human endothelial cells [42]. Hyperinsulinemic induction significantly decreased high molecular weight and total adiponectin levels but increased NF-kB activity in serum treated microvascular endothelial cells [42].

### Lifestyle and circadian disruption

Regarding the contribution of environment in the genesis of resistant hypertension, obesity and T2D, the circadian disruption seems to be an important complicating factor. Disrupted circadian rhythms caused by disturbed sleep-wake cycles, inactivity during the active period, enhanced activity during the rest period and high food consumption might lead to attenuated feeding rhythms, disrupted metabolism and obesity [43]. This lifestyle may cause high parasympathetic output to the viscera leading to obesity, hyperinsulinemia, and hyperlipidemia, or high sympathetic output to the muscle and heart leading to vasoconstriction and hypertension [44]. In addition to the impairment of AI, circadian disruption was found in both groups. Since the genetic background allied to the disruption of the circadian rhythm favors HTN and metabolic disorders, our results are in accordance with the literature: findings in murine models show the strong link between genetic background and circadian rhythm disruption in determining the severity of metabolic disorders [43].

### Hypoadiponectinemia and metabolic disorders

Diabetic patients had greater BMI and lower adiponectin values compared to non-T2D patients. It was also notable that the diabetic group had greater BMI and higher serum triglyceride levels than the non-diabetic group. The association between BMI and hypertriglyceridemia is also in line with the scientific literature [45]. In addition, hypoadiponectinemia *per se *may not be the main issue in metabolic alterations associated with obesity. A harmonized pattern of rhythmic expression of adiponectin by visceral and subcutaneous abdominal adipose tissue seems to be crucial to body fat distribution homeostasis as well as to prevent metabolic alterations associated with obesity [46].

**Figure 4:**
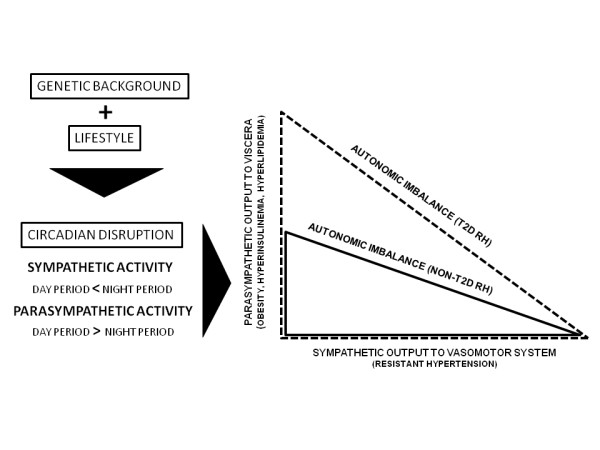
**Circadian disruption and autonomic imbalance and their relationship with vasomotor and visceral disturbances in non-T2D and T2D resistant hypertensive patients**. The circadian disruption maximizes the autonomic imbalance in the development of obesity and T2D in RH.

Based on the above considerations, the imbalance of autonomic function represents a primary defect leading to RHTN and insulin resistance. Moreover, circadian disruption caused by behavior and genetic background disturbs the balance between sympathetic and parasympathetic branches during day and night periods. In our opinion, a higher degree of AI underlies obesity and T2D onset in RHTN compared to non-T2D RHTN (Figure 4).

### Severity of hypertension and autonomic imbalance

However, the significant worsing in AI found in the T2D group is of interest because we considered the null hypothesis of no difference between groups due the severity of hypertension such in RHTN. At first, we presumed that AI could be different in mild to moderate stages of hypertension whilst in RHTN this difference would be strongly attenuated. Since the degree of impairment in cardiac autonomic control is proportional to the severity of hypertension, and not necessarily impaired in mild long-lasting essential hypertension [47], T2D comorbidity would be determinant of AI in mild to moderate hypertension, but not so decisive for AI in RHTN. In fact, Mussalo H et al [47] compared HRV between severe and mild hypertension. However, the severe group was not stratified into non-T2D and T2D subgroups. Once more, in accordance with the clinical trials of our laboratory [48], RHTN proved to have a plural complexity. The AI gap between both groups demonstrated that RHTN *per se *is not an end stage of hypertension disease. Especially in RHTN without T2D, there is still a favorable residual autonomic function which needs to be preserved or enhanced.

### Pharmacologic perspectives for RHTN and T2D treatment

It is important to point out that in cases of T2D comorbidity, a better awareness of specific characteristics of diabetes such as decreased energy expenditure, lipolysis and insulin sensitivity can guide doctors to avoid drugs which can enhance these phenomena such as conventional or non-vasodilating beta-blockers [49]. However, considerable heterogeneity exists within the β-blocker class. Perhaps most important is a variation in adrenergic receptor selectivity; nevertheless, there is also variability in lipophilicity, intrinsic sympathomimetic activity (ISA), membrane-stabilizing action, and vasodilating properties [50]. Carvedilol, for instance, is a highly nonselective lipophilic β-blocker with α_1_-blocking activity and no ISA [51]. Its pharmacologic properties may be associated with a more comprehensive treatment of AI since they can improve insulin sensitivity in T2D patients [52-54]. This mechanism, although very intriguing, needs further support from more complete (possibly longitudinal) studies and opens a new and stimulating field towards pharmacology intervention focusing the improvement of autonomic imbalance in T2D patients in order to optimize glucose metabolism in addition to BP control in RHTN patients.

### Non-pharmacologic treatment of RHTN in real-life settings

There are different methods to measure the circadian rhythm: sleep diaries, polysomnographic recording, actimetry, chronotype identification, body temperature, pre-sleep measures of melatonin secretion, cortisol secretion and activity of clock genes [55]. However, the approach of circadian rhythm may not need all these methods, but simple oriented questions regarding day-night behavior. In addition to the well-known lifestyle factors to be assessed by anamnesis such as obesity, dietary salt intake and alcohol consumption [22], we endorse that the approach of the chronotype of RHTN patients should also be encouraged by all hypertension guidelines.

The circadian disruption found in this study also reinforces the need for lifestyle change in RHTN. Even in asymptomatic obese adults, abnormalities in circadian blood pressure variability and endothelial function exhibited unfavorable cardiometabolic profiles such as elevated high-sensitivity C-reactive protein, fibrinogen, fasting serum glucose and cardiac risk ratios (Total Cholesterol:HDL-cholesterol and LDL-cholesterol:HDL-cholesterol ratios) [56]. A more comprehensive anamnesis over feeding behavior, physical inactivity during the day, hyperactivity during the night (or repose) and duration of sleepiness may help physicians and patients think about strategies to avoid the circadian disruption.

### Limitations of the study

Our main limitation was the recruitment of "true" RHTN patients and then the small sample size is noteworthy. Despite of the small sample size, it was possible to identify greater AI in the T2D subgroup. We assumed that the small sample size of the T2D group is a result of the prevalent exclusion criteria of moderate to severe renal dysfunction, history of coronary artery disease and β-blocker already in use in this population.

### General findings and future perspectives

We concluded that, in spite of circadian disruption, even in RHTN patients there is a residual autonomic function compared to T2D patients. Furthermore, it is clear that the evaluation of BMI, adiponectin and HRV of RHTN patients can reveal the risk of T2D association or future development in this high risk population. However, an appropriate risk matrix to evaluate this prognostic information will demand a longitudinal clinical study with significant greater sample size of RHTN patients.

## Conclusion

Type 2 diabetes comorbidity is associated with greater autonomic imbalance, lower adiponectin levels and greater BMI in RHTN patients. Moreover, similar circadian disruption was also found in both groups indicating the importance of lifestyle behavior in the genesis of RHTN. A better comprehension of the patterns of autonomic imbalance and circadian disruption in RHTN is one more parameter to guide clinicians toward a holistic treatment of hypertension and diabetes.

## List of Abbreviations Used

AI: autonomic imbalance; HTN: hypertension; T2D: type 2 diabetes; HRV: heart rate variability; BMI: body mass index; RHTN: resistant hypertension; SBP: systolic blood pressure; DBP: diastolic blood pressure; EDTA: ethylenediamine tetraacetic acid; LVEF: left ventricle ejection fraction; LVMI: left ventricle mass index; DT: day time; NT: night time; rMSSD: Square root of the mean of the squares of differences between successive RR intervals; SDNN: Standard deviation of all normal RR intervals in 24-hour Holter recording; SDANN: Standard deviation of RR intervals means in all 5-minute segments of 24-hour recording; pNN50: Percentage of differences between successive RR intervals that are greater than 50 ms; FFT: Fast Fourier Transformation; LF nu: low frequency in normalized units; HF nu: high frequency in normalized units; VLF: very low frequency; SD: standard deviation; SEM: standard error of the mean; ANS: autonomic nervous system; OSA: obstructive sleep apnea; ISA: intrinsic sympathomimetic activity; NF-kB: nuclear factor-kappaB.

## Competing interests

LBM and CD are employees of Novartis Biociências S.A. (Brazil).

## Authors' contributions

LBM, LCM and HMJ contributed to the design, analysis, and interpretation of this study. VNF and CD contributed to the collection and critical analysis of clinical and laboratory data. FPSC and MJF contributed to critical analysis of heart rate variability data. FCB contributed to the writing of this manuscript and critical review of the version for submission. All authors have read and approved the final manuscript.
